# *Mycobacterium kansasii* osteomyelitis – a masquerading disease

**DOI:** 10.1099/jmmcr.0.005114

**Published:** 2018-01-02

**Authors:** Kiranmai Bhatt, Krishna Banavathi

**Affiliations:** Royal Stoke University Hospital, Newcastle Road, Stoke-on-Trent ST4 6QG, UK

**Keywords:** *Mycobacterium kansasii*, non tuberculous mycobacteria, soft tissue infection, bone infection, osteoarticular infection

## Abstract

**Introduction:**

Non-tuberculous mycobacteria (NTM) are environmental bacteria capable of causing an opportunistic myriad of infections. *Mycobacterium kansasii*, one such NTM, is responsible for causing pulmonary disease in immunocompromised patients. Rare extrapulmonary manifestations such as lymphadenitis, osteoarticular manifestations, and skin and soft tissue infections are also observed.

**Case presentation:**

Here, we report an unusual case of sternoclavicular joint and elbow joint infection with *M. kansasii* in a relatively immunocompetent patient. Histopathology did not show classic granulomas and mycobacterial infection was not initially considered as a possibility. However repeat biopsies were sent for mycobacterial cultures which then grew *M. kansasii*.

**Conclusion:**

Diagnosis of *M. kansasii* in such cases can be difficult and culture-positive results may not necessarily imply positive diagnosis as they can be environmental contaminants. Furthermore, *M. kansasii* can cause infections without the characteristic granuloma formation, which can further complicate tissue diagnosis. This underlines the importance of ensuring that tissue samples obtained are cultured for mycobacteria.

## Introduction

*Mycobacterium kansasii* is a non-tuberculous, slow-growing mycobacteria first described in 1953 as causing infections in humans [1]. It is widely spread in the environment, commonly in aquatic environments, tap water and soil [2]. Infection is thought to be transmitted by the aerosol route and occurs primarily in immunocompromised individuals. While extrapulmonary *M. kansasii* infection is rare, its incidence in the UK is higher than the global average [3]. In contrast to other non-tuberculous mycobacteria (NTM) infections, *M. kansasii* is rarely associated with lymphadenopathy. Additionally, skin and soft tissue infections are not very common extrapulmonary manifestations of *M. kansasii* infection, with osteomyelitis being even rarer. We report a case of skin and soft tissue infection with progression to osteomyelitis with *M. kansasii* infection in an immunocompetent individual. To our knowledge this is the first case report of sternoclavicular joint and elbow joint infection with *M. kansasii*.

## Case report

**Fig. 1.**
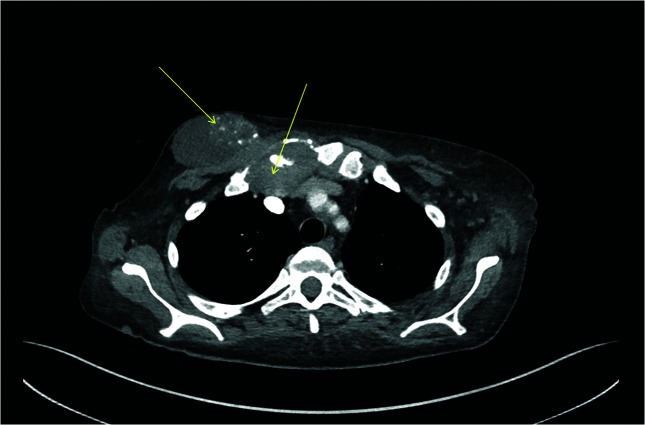
Computed tomography (CT) thorax showing the sternoclavicular mass as indicated by the two arrows.

**Fig. 2.**
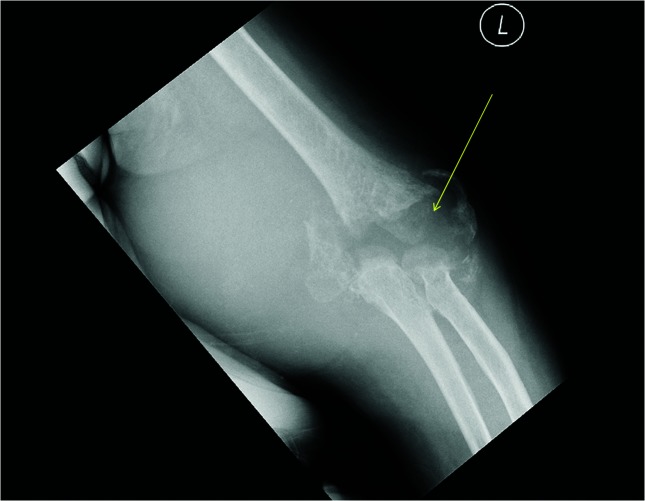
X-ray of the left elbow joint shows osteomyelitis and destruction of the joint as indicated by the arrow.

A 50-year-old Caucasian female presented initially with a spontaneously occurring, non-tender, right anterior chest wall soft tissue cystic-like mass in late 2014 (Fig. 1). Her past medical history included Waardenburg syndrome (a genetic condition associated with hearing loss and changes to hair skin and eye pigmentation), well-controlled non-insulin-dependent diabetes, learning difficulties and she had 15 surgeries performed on her deformed feet. She also suffered from sideroblastic syndrome since 2002 for which she was receiving blood transfusions three times a week with well-controlled iron overload. A month after developing the right anterior chest wall mass, she developed a non-tender swelling in her left elbow which got progressively larger and uncomfortable (Fig. 2). She was seen by both orthopaedic and rheumatology teams, who after reviewing the images suspected that the swelling could be due to an inflammatory reaction rather than infection. She was therefore started on a reducing dose of 30 mg oral prednisolone which she took intermittently for a year. During this time her swelling remained non-tender, however towards the beginning of January 2016 she presented with pain in her elbow joint. Repeat imaging showed worsening of the left elbow joint mass and an enlarging right sternoclavicular mass. She was referred to a specialist centre for biopsy of the right sternoclavicular joint mass for suspected lymphoma. In April 2016, multiple biopsies of her right sternoclaicular joint showed necrotic material and inflammatory cells along with granulation tissue suggestive of osteomyelitis. As no evidence of granulomas was seen, tissue samples were not sent for any mycobacterial cultures. She was started on oral flucloxacillin 1 g QDS to be taken for 4 weeks. At the beginning of May 2016, while on flucloxacillin, she became unwell with signs of sepsis and her left elbow swelling had started to ulcerate. Chest X-ray on admission showed infective changes to the bases of both lungs. She was commenced on IV co-amoxiclav. Microbiology samples from previous biopsies in another hospital showed *Pseudomonas pseudoalcaligenes* and scanty growth of *Staphylococcus epidermis*. Blood cultures grew *Enterobacter cloacae*. The patient was discussed in the orthopaedic multidisciplinary team (MDT) meeting and a week after admission she was taken to theatre for left elbow debridement which showed a completely destroyed elbow joint with frank pus and necrotic tissue. At the same time, incisional biopsy of the right sternoclavicular joint mass showed frank fatty pus. Multiple specimens were obtained from both sites and sent for histology, microbiology and mycobacterial cultures. Post-surgery the patient was commenced on IV vancomycin with no improvement to her condition. Microbiological results showed scanty growth of skin organisms such as *S. epidermi*s. Multiple samples processed for mycobacterial cultures of both right sternoclavicular joint tissue and left elbow were positive for *M. kansasii*. The identification of the isolate was done by the reference laboratory by using molecular methods. Before mycobacterial culture results were available, the patient became unwell with progressive cough, pyrexia and shortness of breath. She was commenced on IV meropenem along with IV vancomycin. She progressively became septic and suffered a fatal cardiac arrest a week after debridement. In view of this we were unable to commence *M. kansasii* treatment.

## Discussion

NTMs were first identified as pulmonary pathogens in the 1950s by Runyon [4]. Following this, increasing numbers of NTMs have been recognized as emerging pathogens, especially in immunocompromised individuals. NTMs can involve multiple organs including bones, though osteomyelits caused by an NTM is very rare. There are case reports of *M. kansasii* causing osteomyelitis but these are usually in immunocompromised patients [5–10]. In this case, the patient was not severely immunocompromised, and the source of entry of the pathogen could have been the numerous foot surgeries she had, or via the pulmonary or gastrointestinal route. Her diabetes, in spite of being non-insulin dependent, along with sideroblastic anaemia could have contributed to a possible compromised immune system making her more susceptible to NTM infection. It is likely that in immuncompetent patients, mucosal surfaces of the respiratory or gastrointestinal tract could be invaded or colonized and the bacteria are engulfed by resident macrophages. Mycobacteria evade killing in a macrophage by preventing phagosome–lysosome fusion [11]. Through complex mechanisms via nitric oxide signalling pathways, these macrophages come in contact with the site of bone remodelling where NTMs are released causing local osteomyelitis [12–14]. Dissemination of NTMs resulting in osteomyelitis is a very complex progression and not well understood.

Diagnosis of NTM infections is very difficult as there is no single diagnostic test available. This could also explain why the rates of osteomyelitis due to NTMs are so low. In this patient, mycobacterial infection was not considered as a possible diagnosis, and also during the initial biopsy due to lack of granulomas, samples were not sent for mycobacterial culture. It is important to appreciate that infection with *M. kansasii* can result in any of the five to six types of reactions seen in two different studies. Five types of reactions seen in one study were: (1) abscesses; (2) granulomas without giant cells but with large areas of central eosinophilic necrosis with numerous neutrophils and nuclear debris; (3) well-organized granulomas without giant cells or necrosis, but with a mononuclear cell infiltrate; (4) areas of eosinophilic granular necrosis with scattered clusters of epithelioid histiocytes; and (5) spindle-cell proliferations with scattered clusters of neutrophils [15]. A sixth type of reaction, sheets of foamy histiocytes containing acid-fast bacilli (AFB), was observed in another study [16]. In this patient, initial histology showed non-graulomatous necrotic material suggestive of a either a type 4 or 5 reaction. Unfortunately, due to lack of knowledge, the possibility of myocbacterial infection was not considered and hence not investigated. Dissemination of *M. kansasii* can be an indolent process and difficult to diagnose.

In summary, the route of entry for *M. kansaii* in our patient could have been either related to her foot surgeries or a non-traumatic route via the respiratory/gastrointestinal tract. It is imperative to consider mycoabacterial infections as a differential when assessing soft tissue non-tender masses in both immunocompromised and immunocompetent individuals. The absence of classic granulomas, as seen in this case, should not be used to rule out mycobacterial infection.
